# In silico prediction of lncRNA function using tissue specific and evolutionary conserved expression

**DOI:** 10.1186/s12859-017-1535-x

**Published:** 2017-03-23

**Authors:** Umberto Perron, Paolo Provero, Ivan Molineris

**Affiliations:** 1 Department of Molecular Biotechnology and Health Sciences, University of Turin, via Nizza 52, Torino, 10126 Italy; 2 Center for Translational Genomics and Bioinformatics, San Raffaele Scientific Institute, via Olgettina 60, Milan, 20132 Italy

**Keywords:** lncRNA, Functional prediction, Disease gene prediction, Coexpression

## Abstract

**Background:**

In recent years long non coding RNAs (lncRNAs) have been the subject of increasing interest. Thanks to many recent functional studies, the existence of a large class of lncRNAs with potential regulatory functions is now widely accepted. Although an increasing number of lncRNAs is being characterized and shown to be involved in many biological processes, the functions of the vast majority lncRNA genes is still unknown. Therefore computational methods able to take advantage of the increasing amount of publicly available data to predict lncRNA functions could be very useful.

**Results:**

Since coding genes are much better annotated than lncRNAs, we attempted to project known functional information regarding proteins onto non coding genes using the guilt by association principle: if a gene shows an expression profile that correlates with those of a set of coding genes involved in a given function, that gene is probably involved in the same function. We computed gene coexpression for 30 human tissues and 9 vertebrates and mined the resulting networks with a methodology inspired by the rank product algorithm used to identify differentially expressed genes. Using different types of reference data we can predict putative new annotations for thousands of lncRNAs and proteins, ranging from cellular localization to relevance for disease and cancer.

**Conclusions:**

New function of coding genes and lncRNA can be profitably predicted using tissue specific coexpression, as well as expression of orthologous genes in different species. The data are available for download and through a user-friendly web interface at www.funcpred.com.

**Electronic supplementary material:**

The online version of this article (doi:10.1186/s12859-017-1535-x) contains supplementary material, which is available to authorized users.

**Electronic supplementary material:**

The online version of this article (doi:10.1186/s12859-017-1535-x) contains supplementary material, which is available to authorized users.

## Background

In recent years long non coding RNAs (lncRNAs) have been the subject of increasing interest. Although some lncRNAs such as Xist [1] and H19 [2] were discovered decades ago, it was only recently established that mammalian genomes encode several thousands lncRNAs [3]. Their low sequence conservation across model organisms and low expression levels have led some to postulate that many lncRNAs could arise from low fidelity RNA polymerase activity, and that this spurious activity is of little significance [4]. However, thanks to many recent functional studies, the existence of a large class of lncRNAs with potential regulatory function is now widely accepted [5, 6]. Although an increasing number of lncRNAs is being characterized and shown to be involved in many physiological and pathological biological processes, the function of the vast majority of lncRNA genes is still unknown. There is therefore a need for tools that are able to systematically infer a function for large numbers of lncRNAs starting from currently available data such as gene expression. Computational investigation of lncRNAs function is challenging due to the fact that many lncRNA do not contain conserved sequence motifs [3], which makes it difficult to infer potential functions of lncRNAs based on their sequences alone. Coexpression relationships represent an extremely rich source of information, potentially relevant for functional annotation. Indeed, it has been shown extensively that functionally interacting genes tend to show similar expression profiles [7, 8] and gene expression data were used in several works devoted to lncRNA function prediction. More specifically Liao and colleagues, starting from microarray expression profiles, built a coding-non coding network which was then used to infer probable functions for lncRNAs based on topological characteristics [9]. Another work by Guo et al. developed a lncRNA function predictor that works by integrating gene expression data and protein interaction data [10]. Most recently, Jiang and colleagues [11] based their strategy on expression correlation between lncRNAs and protein-coding genes across several human tissues without considering tissue specificity.

Individual genes of multicellular organisms can participate in different transcriptional programs, operating at scales as different as single-cell types, tissues, organs, body regions or the entire organism. We and others have shown in the past that systematic analysis of tissue-specific coexpression is a powerful strategy to dissect functional relationships among genes that emerge only in particular tissues or organs [12]; to our knowledge this strategy has never been applied to lncRNA fuctional prediction. Moreover, the probability for two genes to be functionally correlated is remarkably higher when they are coexpressed in more than one species (conserved coexpression) [12, 13].

The GTEX consortium has made available a dataset of about three thousands samples of human gene expression data in multiple tissues [14] while Necsulea and coworkers [15] curated a collection of about two hundred samples across 10 species. These data are obtained with RNA-seq technologies that detect lncRNAs as well as protein-coding mRNAs. Thus it is now possible to develop methodologies able to perform functional annotation of lncRNAs that take into account the tissue-specificity of gene function and that integrate coexpression of orthologous genes in several species. In this paper we present a novel methodology to perform in-silico functional annotation of genes. In particular we aim to predict the functions of lncRNAs on the basis of their coexpression with known protein-coding genes in many tissues and species.

## Methods

### RNA-seq dataset

We used two RNA-seq datasets, the first to evaluate phylogenetically conserved coexpression, the second to evaluate tissue-specific coexpression. The first dataset consists of 185 RNA-seq samples across 10 species (human, chimpanzee, gorilla, orangutan, macaque, mouse, opossum, platypus, chicken and frog) and 8 organs (cortex or whole brain, cerebellum, heart, kidney, liver, placenta, ovary and testes) previously published by Necsulea et al. [16]. In this dataset about 22000 protein-coding genes and 5400 lncRNAs are profiled. We downloaded lncRNA orthologous families and normalized gene expression levels for lncRNAs and protein-coding genes from the supplementary material of [16]. The second dataset consists of 2923 RNA-seq samples collected by the GTEX consortium [14]. We used the more coarse-grained sample annotation provided to sort all the samples in 30 tissues. In this dataset about 19500 protein-coding genes and 7000 lncRNA are profiled.

### Homology relations

To reconstruct homology relationships we used both orthologous genes downloaded from ENSEMBL and Necsulea et al.’s lncRNAs families. We also included one-to-many homology relationships.

### Gene annotation sources

#### Gene Ontology

We used two controlled vocabularies to annotate genes: Gene Ontology (GO) [17] downloaded from ENSEMBL and Disease Ontology (DO) [18]. We also make use of literature-mined disease-gene associations from DISEASE [19]. For both GO and DO we calculated how many genes were annotated for each term and then we used only those terms that had between 4 and 1600 genes, discarding the poorly informative and very generic terms like “cytoplasm” and “metabolism” or terms too specific to be suitable for our model. Finally, we also used the Generic GO slim developed by GO Consortium [20].

#### MSigDB gene sets collections

We considered three collections of gene sets from MSigDB [21]: 1) Hallmark (MSigDBh), that collect coherently expressed signatures that represent well-defined biological states or processes 2) MSigDBc2, that collect the curated gene sets from online pathway databases, publications in PubMed and knowledge of domain experts, 3) the oncogenic signatures collection MSigDBc6, defined from microarray gene expression data from cancer gene perturbations.

### Functional prediction score

Given a set of RNAseq samples and the quantification of gene expression on each sample, let *P*(*a*,*b*) be the Pearson correlation coefficient of the expression profiles of the genes *a* and *b*. In the following, we define coexpression networks as the complete undirected networks that have genes as nodes and whose links (*a*,*b*) are weighted using *P*(*a*,*b*). Let *R*(*a*) be the list of *P*(*a*,*b*) computed against all genes *b* keeping gene *a* fixed, sorted on the value of the Pearson coefficient. The position of *b* in the *R*(*a*) list is the rank *R*(*a*,*b*), in the following we always use a normalized rank, namely $r(a,b)=\frac {R(a, b)}{\#R(a)}$ where *#* *R*(*a*) is the length of the list.

Given a gene *a* and a set of genes *G*_*k*_ annotated to some keyword *k*, we computed the functional prediction score (FPS) related to *a* and *k* as the logarithm of the geometric mean of the ranks of all genes annotated to *k* in the ranked list of *a*:

1 $$ \text{FPS}(a, G_{k}) = \frac{1}{\#G_{k}}\sum_{i\in G_{k}}\log\left(r(a,i)\right)  $$

where *#* *G*_*k*_ is the number of gene annotated to *k*. This score is inspired by the rank-product algorithm proposed by Breitling and colleagues [22].

The Pearson correlation of two genes *a* and *b* is symmetric *P*(*a*,*b*)=*P*(*b*,*a*), on the contrary the rank of the correlation is not symmetric: *r*(*a*,*b*)≠*r*(*b*,*a*). In the context of regulatory network inference a mutual rank transformation of the correlation has been proposed in order to obtain a measure that maintains the properties of the rank but is symmetric. We evaluated different procedures to transform the Pearson rank in a symmetric measure: the geometric mean proposed by Obayashi et al. [23], the standard average and the maximum of the two different ranks. None of these transformations led to significant improvements in prediction performance.

### Identification and representation of GO terms typical of lincRNA

**Fig. 1:**
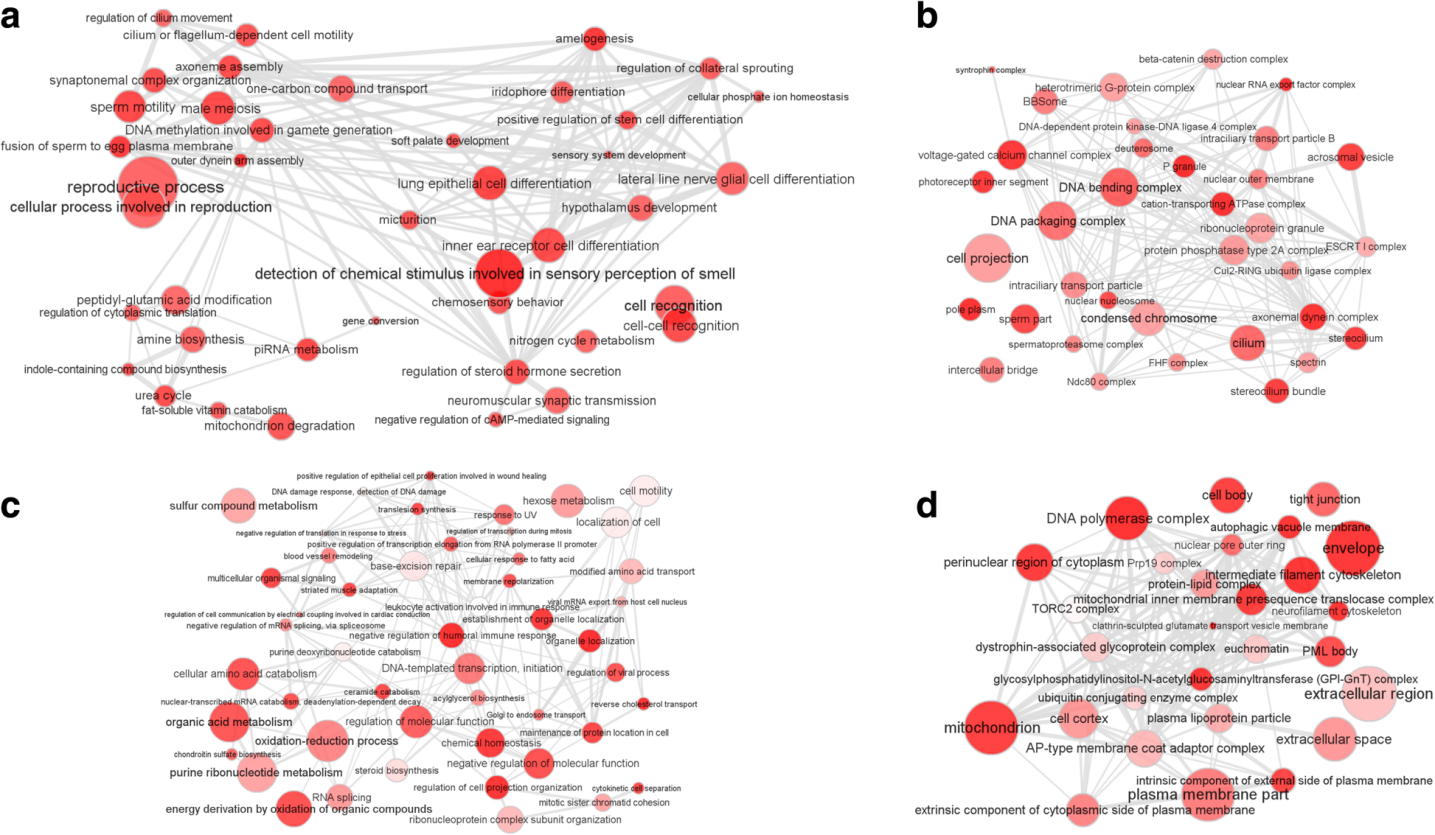
Summarized representation of predicted GO terms that are more typical of lincRNAs than PCGs and vice versa. **a** GO biological process terms typical of lncRNAs; **b** GO cellular component terms typical of lncRNAs; **c** GO biological process terms typical of PCGs; **d** GO cellular component terms typical of PCGs. *Bubble color* represents how much the term is specific for PCGs or lincRNAs (brighter is more specific); *bubble size* indicates the frequency of the GO term in the whole GO database. Highly similar GO terms are linked by edges in the graph, where the line width indicates the degree of similarity

We intended to identify predicted terms that are more typical of lincRNAs (long intergenic non-coding RNA), a subset of lncRNAs, than PCGs (or vice versa). For this analysis we focused on lincRNAs to avoid bias that couls be introduced if we considered together all lncRNAs since they also include pseudogenes. For each GO term *k* we ranked all genes *g* according to the FPS(*g*,*k*), then we compared the ranks of lincRNAs and PCGs with the Wilcoxon rank-sum test. To choose the 100 most typical predicted term to be further analyzed we computed the difference of the median rank-transformed FPS between lincRNAs and PCGs, then we selected the lowest 100 as lincRNAs-related and the best 100 as the PCG-related. We used REVIGO [24] to summarize the predicted GO keyword lists and to plot the Fig. 1. This tool uses a clustering algorithm that relies on semantic similarity measures to select a representative subset of the terms. The bubble color saturation represents the absolute value of the difference between the median rank-transformed FPS of lincRNAs and the median rank-transformed FPS of PCGs for that term. Highly similar GO terms are linked by edges in the graph, where the line width indicates the degree of similarity. Finally, bubble size is a measure of how frequently the term appears in the whole GO database.

### Validation on coding genes

In order to validate the performance of our method, we first considered only genes already annotated with GO vocabulary (those are all PCGs) and we performed a ROC analysis. For each ontology term *k* a gene *g* is considered positive if it is annotated to that term, and negative if it is not. A ROC curve is computed based on this binary classifications of all genes and the FPS. We adopted a leave-one-out procedure: if *g*∈*G*_*k*_, when computing FPS(*g*,*k*) we do not use as usual *G*_*k*_ but we exclude *g* from *G*_*k*_ and consider instead $G^{\prime }_{k} = G_{k}-\left \lbrace g\right \rbrace $. For all keywords *k* the number of positive genes *#* *G*_*k*_ is much less than the number of negatives, therefore in the ROC analysis we considered not all the negative genes but, for each *k* independently, only a randomly chosen subset of size *#* *G*_*k*_, this procedure is the same used by Guo et al. [10]. Due to the hierarchical structure of the GO vocabulary, the GO keywords associated to a gene are highly non-independent: if a gene *g* is annotated with a certain keyword *k* then *g* is also related to each keyword *k* ^′^ that is an ancestor for *k*. Since the ROC analysis requires independent observed events, we derive from the standard GO a “non redundant” version in which each gene is associated to only one term.

### Non redundant version of GO (GOnrBP)

First of all we chose only the keywords belonging to the biological process ontology (BP), then for a given gene *g* we discarded all keywords but the smallest keyword *k*, i.e. most specific among those associated with *g*, provided that *#* *G*_*k*_>5. This procedure removes the dependence between gene-function annotations that is due to the hierarchical structure of GO: indeed in GOnrBP *G*_*k*_∩*G*_*h*_=*∅* ∀*k*≠*h*.

### Validation on lncRNA

#### Gene ontology

Starting from all the genes in lncRNAdb [25] we considered the genes that: a) have an ENSEMBL gene identifier reported in lncrnadb version 2.0, b) are expressed in the GTEx database, c) are annotated as lncRNA by Ensembl, d) are annotated with a known function by lncrnadb. The resulting set is composed by 37 genes. Each of those genes was manually annotated with one term from the generic GO slim developed by GO consortium starting from the description reported in lncrnadb (Table 1). Then we computed the FPSs for these 37 genes and all GO terms using all GTEx samples together. We finally compared the FPSs related to the selected GO keywords with all other keywords in GO using the Wilcoxon rank-sum test.

**Table 1:** The table displays a set of 37 genes that were manually annotated with one term from the generic GO slim starting from the functional description reported in lncRNAdb

ENSG	GOslim	ENSG	GOslim
ENSG00000130600	GO:0009790	ENSG00000230590	GO:0000228
ENSG00000130600	GO:0040007	ENSG00000230590	GO:0005694
ENSG00000130600	GO:0000988	ENSG00000231265	GO:0048870
ENSG00000130600	GO:0006412	ENSG00000231265	GO:0016301
ENSG00000130600	GO:0008283	ENSG00000236790	GO:0048856
ENSG00000153363	GO:0008219	ENSG00000241684	GO:0048870
ENSG00000153363	GO:0040007	ENSG00000241684	GO:0000988
ENSG00000176840	GO:0008219	ENSG00000241743	GO:0003677
ENSG00000177410	GO:0040007	ENSG00000241743	GO:0000228
ENSG00000177410	GO:0008283	ENSG00000241743	GO:0005694
ENSG00000177410	GO:0030154	ENSG00000244306	GO:0030154
ENSG00000204092	GO:0008283	ENSG00000244306	GO:0048870
ENSG00000204092	GO:0007049	ENSG00000244306	GO:0006397
ENSG00000204092	GO:0003723	ENSG00000244306	GO:0007165
ENSG00000214548	GO:0040007	ENSG00000245532	GO:0030674
ENSG00000214548	GO:0021700	ENSG00000245532	GO:0005634
ENSG00000214548	GO:0030154	ENSG00000245532	GO:0043234
ENSG00000214548	GO:0000988	ENSG00000245532	GO:0005198
ENSG00000214548	GO:0006259	ENSG00000245532	GO:0065003
ENSG00000223403	GO:0000988	ENSG00000245910	GO:0006412
ENSG00000223403	GO:0009790	ENSG00000245910	GO:0005840
ENSG00000223403	GO:0040007	ENSG00000247556	GO:0009790
ENSG00000223403	GO:0048856	ENSG00000247556	GO:0048646
ENSG00000223573	GO:0030154	ENSG00000247844	GO:0008283
ENSG00000223573	GO:0006397	ENSG00000247844	GO:0048870
ENSG00000223573	GO:0003723	ENSG00000248323	GO:0008283
ENSG00000223573	GO:0003729	ENSG00000249669	GO:0030154
ENSG00000223850	GO:0008283	ENSG00000249669	GO:0000988
ENSG00000223850	GO:0006397	ENSG00000249859	GO:0005578
ENSG00000224177	GO:0005856	ENSG00000249859	GO:0008283
ENSG00000224177	GO:0005198	ENSG00000249859	GO:0008219
ENSG00000225127	GO:0040007	ENSG00000249859	GO:0030154
ENSG00000225127	GO:0021700	ENSG00000250366	GO:0009790
ENSG00000225407	GO:0009790	ENSG00000250366	GO:0048856
ENSG00000225407	GO:0000988	ENSG00000251002	GO:0006259
ENSG00000225407	GO:0005634	ENSG00000251002	GO:0005634
ENSG00000225407	GO:0000228	ENSG00000251002	GO:0002376
ENSG00000225407	GO:0051276	ENSG00000251164	GO:0008283
ENSG00000225506	GO:0030154	ENSG00000253352	GO:0008219
ENSG00000225783	GO:0006397	ENSG00000253352	GO:0000988
ENSG00000225783	GO:0030154	ENSG00000253352	GO:0000228
ENSG00000225783	GO:0048856	ENSG00000253438	GO:0006950
ENSG00000225783	GO:0003723	ENSG00000253438	GO:0006412
ENSG00000225783	GO:0030154	ENSG00000253438	GO:0006259
ENSG00000229140	GO:0008283	ENSG00000255733	GO:0002376
ENSG00000229140	GO:0030154	ENSG00000258399	GO:0009790
ENSG00000229140	GO:0008219	ENSG00000258399	GO:0000988
ENSG00000229807	GO:0030234	ENSG00000258399	GO:0000228
ENSG00000229807	GO:0003677	ENSG00000258609	GO:0030154
ENSG00000229807	GO:0000228	ENSG00000258609	GO:0008219
ENSG00000229807	GO:0005694		

#### Disease ontology

As before we manually annotated, this time to DO terms, the lncRNA-disease associations collected in the LncRNA disease database [26]. In this process we only took into consideration those lncRNA genes that are both annotated with some term in in LncRNAdisease and are contained in Ensembl (see Additional file 1).

#### LncRNA implicated in cancer

We analyzed the lncRNA that are reported by Khurana et al. [27] to carry oncogenic mutation in cancer. Among those we selected only the ones that have an ENSEMBL identifier and are expressed in the GTEx dataset (MYCNUT, BRAFP1, PTENP1 and TUSC7). Using FPSs computed on GTEx combined expression dataset we computed, for each given function *k* reported in MSigDBh, a Wilcoxon rank-sum statistics comparing {FPS(*g*,*k*) ∀*g*∈{MYCNUT, BRAFP1,PTENP1,TUSC7}} whit {FPS(*g*,*h*) ∀ *g*∈{MYCNUT, BRAFP1, PTENP1,TUSC7}, ∀ *h*≠*k*}.

The *P*value of the test measures the probability that, for a given set of genes (composed by four genes in this case) a certain keyword *k* ranks better than other keywords *h* for these genes together.

### Logistic model for tissue-specific evaluation

We have an observable *O*(*g*,*k*) for each gene-keyword pair (*g*,*k*), *O*(*g*,*k*)=1 if *g*∈*G*_*k*_ 0 otherwise. As predictors we used FPS_*TS*_(*g*,*k*) computed on each of the 30 tissue-specific coexpression networks (TS), plus FPS_*AS*_(*g*,*k*) computed on the aggregate coexpression network.

Here we intended to demonstrate that FPSs computed on tissue-specific networks can significantly improve the functional predictions. Because of this we only focus on genes expressed in all tissues and thus discard those genes that are not expressed in one or more tissue.

Statistical evaluations of the models are guaranteed to be correct only if the observations are independent. Due to the hierarchical structure of GO this is not the case; we therefore employed the custom-built GOnrBP version described previously. However, we have observed no significant difference in results when using GOnrBP or the standard GO version.

As before we randomly down-sampled the negatives for each *k* in order to have a balanced dataset. Overfitting is not a concern since we are using only 32 parameters: the intercept, the coefficients associated to 30 TS plus that associated to AS and have more than 150,000 cases. The area under the ROC curves (AUCs) is calculated using the predicted probability resulting from the fitted models as score.

### Comparison with other methods

**Fig. 2:**
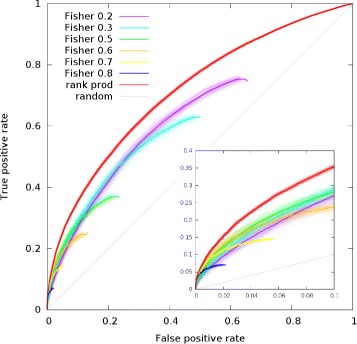
ROC curve comparing the performance of our approach (rank prod) and the standard enrichment method (Fisher). Different cutoffs are applied to the Pearson correlation that measures the gene coexpression. The *little box* is a zoom of the region of small FPS. *Blurred thick curves* result from the overlap of semi-transparent curves derived by 100 random sampling for each cutoff and our method. *Dark thin line* result from the averaging of such data

We perform a ROC analysis comparing our approach with an enrichment approach based on Fisher exact test [11]. Jiang and colleagues used a dataset consisting of around 60 samples obtained from 22 human tissues and 3 human cell-lines; since they do not use phylogenetic conservation in the comparison we used only human samples. The dataset that we use is statistically richer (about 3000 samples in 30 tissues) thus, for a safer comparison, we applied the same procedure described by Jiang et al. to our dataset. Moreover they apply a fixed cutoff of 0.9 on Pearson coefficient: this cutoff is probably optimized for the dataset they used and possibly not optimal for the new one, thus we performed the comparison using different cutoffs ranging from 0.9 to 0.2. We then applied a leave one out and down-sampling procedure for negative gene-GO associations as described in the previous section. Finally we compared our FPS(*g*,*k*) with the enrichment *E*(*g*,*k*) measured as

$$E(g,k)=\frac{xN}{nM} $$

were *N* is the total number of annotated genes whose expression is detected in at least one sample, *M*=*#* *G* *k*′ is the number of such genes annotated to the given function, *n* is the number of genes that show a correlation above the cutoff (connected genes in the unweighted coexpression network) and *x* is the number of connected genes annotated to the function. In the same scenario we also evaluated as a score the *p*-value of Fisher exact test and we have not seen significant differences in performance. We also took into account the fact that the number of genes associated to GO keywords can vary from few units to thousands. As before we produced 100 random samples taking only one gene at random (positive or negative) for each keyword in each sample. With this bootstrap procedure we also empirically evaluated the variance of the ROC (see Fig. 2).

## Results

We computed FPSs using together all samples collected by the GTEx consortium. /textcolorredOur approach can predict the function of any kind of expressed gene, in particular we focused on two important gene biotypes, namely protein coding genes (PCGs) and long intergenic non coding RNA genes (lincRNAs), and observed that the annotations we predicted for lincRNAs genes and PCGs are different. In fact 88% of GO biological process terms and 90% of the GO cellular component show different FPSs for the two gene classes (Wilcoxon rank-sum test *P*value <0.05 after multiple testing correction). Then we looked at the 100 cellular localization that are predicted to be more typical of lincRNAs than PCGs and we found terms such as: DNA bindig complex, DNA packaging complex, pole plasm, P granule. This is consistent with the fact that many lincRNA are probably involved in gene regulation and chromatin remodeling. We also found somewhat unexpected localization terms as voltage-gated calcium channel complex or axonemal dynein complex. Looking at the biological processes predicted to be typical of lincRNA we found as expected many differentiation and development processes (e.g. positive regulation of stem cell differentiation, hypothalamus development) or regulatory processes (e.g. DNA methylation involved in gamete generation, piRNA metabolism) but also some unexpected processes like cell recognition and sperm motility (see Fig. 1). Predicted annotation data for PCGs, lincRNA and genes belonging to other classes of lncRNA are available through our web interface (http://www.funcpred.com).

### Validation on coding genes

#### Tissue specific coexpression

Beside FPS computed on all GTEx samples together, we considered FPSs computed on tissue-specific coexpression networks that consider separately samples coming from different tissues. To investigate if the integration of single-tissue FPS and all-sample FPS can improve substantially the performance of our algorithm, we built multivariate logistic models that use 30 different tissue-specific predictors (TSs) plus one predictor coming from all tissues together (AS). For each gene *g* and keyword *k* we construct a response variable equal to 1 if *g* is associated to *k*, 0 otherwise. We fit four types of models, depending on the considered predictors:

1. one single TS (univariate),
2. AS only (univariate),
3. two predictors (AS plus one of the TSs, bivariate),
4. AS plus all TSs (full model, multivariate).

As reported in Table 2 each TS alone is a significant predictor of gene functions and, as expected, AS alone performs better than any other TS. Considering models of type 3 we can see that, taken separately, each TS but one improves significantly the prediction when integrated with AS (*P*value <1*e*−16). The tissue that does not give further information with respect to all-sample is salivary gland, which is also the tissue with the lowest number of sample in the GTEx dataset (6 samples). Fitting a model with all the predictors together we observe an AUC of 0.85, compared to 0.77 that we obtain using only the AS predictor. The full model is therefore better than any model of type 3 but not all the TS have significant log(odds). Performing sequential analysis of deviance the model does not significantly improve after the inclusion of the best 17 TS as predictors (Table 3).

**Table 2:** Log(ods) and relative *P*values associated to different tissues in logistic models

		Univariate			Bivariate			Multivariate	
Predictor	Samples	Log(odds)	AUC	*P*value	Log(odds)	AUC	*P*value	Log(odds)	*P*value
Adipose_Tissue	159	–3.9	0.75	<1e-256	–1.7	0.8	2.4e-214	–0.69	1e-11
Adrenal_Gland	52	–3.6	0.72	<1e-256	–1.7	0.79	5.7e-149	–0.49	1.2e-07
Bladder	11	–1.9	0.65	7.8e-206	–0.59	0.77	3.1e-24	–0.05	0.53
Blood	245	–3.4	0.75	<1e-256	–1.2	0.79	2.6e-115	–0.33	0.00018
Blood_Vessel	263	–4.2	0.75	<1e-256	–1.6	0.8	2.5e-198	–0.18	0.11
Brain	357	–3.4	0.72	<1e-256	–1.6	0.79	3.5e-108	–0.32	0.00029
Breast	66	–3.2	0.71	<1e-256	–1.2	0.79	5.5e-131	–0.03	0.76
Cervix_Uteri	9	–2.5	0.65	5.8e-290	–0.85	0.78	4.1e-42	–0.16	0.1
Colon	74	–3.4	0.71	<1e-256	–1.2	0.79	2.3e-124	–0.25	0.018
Esophagus	227	–3.8	0.74	<1e-256	–1.4	0.79	1.1e-155	–0.29	0.0073
Fallopian_Tube	6	–1.8	0.62	3.1e-182	–0.76	0.77	1e-16	–0.01	0.93
Heart	133	–4.1	0.78	<1e-256	–1.7	0.81	8.5e-298	–0.64	1.7e-10
Kidney	8	–2.2	0.74	<1e-256	–0.89	0.79	1.7e-119	–0.47	5.6e-16
Liver	34	–3.3	0.73	<1e-256	–1.4	0.79	8.5e-166	–0.42	1.3e-06
Lung	133	–3.8	0.74	<1e-256	–1.5	0.79	2.4e-188	–0.28	0.0061
Muscle	157	–3.9	0.78	<1e-256	–1.7	0.81	2.4e-294	–0.98	2.6e-27
Nerve	114	–3.7	0.72	<1e-256	–1.4	0.79	4.2e-148	0.12	0.29
Ovary	35	–2.8	0.68	<1e-256	–1.2	0.78	3.6e-83	–0.08	0.4
Pancreas	65	–3.8	0.79	<1e-256	–2	0.82	<1e-256	–1.1	2.5e-43
Pituitary	22	–1.8	0.66	7.3e-261	–0.69	0.78	4.1e-47	–0.08	0.33
Prostate	42	–3.1	0.7	<1e-256	–1.1	0.78	1.9e-92	0.04	0.7
Salivary_Gland	5	–0.46	0.6	2.9e-27	0.09	0.77	0.21	0.37	2.3e-08
Skin	322	–3.7	0.72	<1e-256	–1.6	0.79	1.5e-129	–0.57	7.4e-10
Small_Intestine	17	–2.5	0.66	<1e-256	–0.81	0.78	6.6e-40	–0.03	0.76
Spleen	34	–2.7	0.67	<1e-256	–1	0.78	5.4e-72	0.03	0.7
Stomach	81	–3.7	0.74	<1e-256	–1.6	0.79	1e-152	–0.23	0.028
Testis	60	–3	0.67	<1e-256	–1.2	0.78	6.2e-78	–0.35	0.00012
Thyroid	120	–3.6	0.72	<1e-256	–1.4	0.79	1.1e-140	0.19	0.071
Uterus	36	–2.7	0.68	<1e-256	–0.99	0.78	9.1e-70	0.06	0.54
Vagina	34	–3	0.7	<1e-256	–1.2	0.78	1.4e-93	0.12	0.25
AS	2921	–3.7	0.77	<1e-256				–1.2	5.9e-51

Bivariate models include two predictors: the indicated TS plus AS, the AUC is relative to the entire model. The multivariate model include all the predictors, in this case the AUC is 0.85

**Table 3:** Sequential analysis of deviance (anova): it sequentially compares the smaller model with the next more complex model by adding one variable (TS) in each step

Predictor	Df	Deviance	Resid. Df	Resid. Dev	Pr(>Chi)
All tissues	1	5534.7	23920	24919	< 2.2e-16***
Pancreas	1	1694.8	23919	23224	< 2.2e-16***
Muscle	1	617.6	23918	22606	< 2.2e-16 ***
Kidney	1	131.7	23917	22475	< 2.2e-16***
Adipose Tissue	1	208.0	23916	22267	< 2.2e-16***
Heart	1	124.2	23915	22142	< 2.2e-16***
Skin	1	79.4	23914	22063	< 2.2e-16***
Salivary Gland	1	27.6	23913	22035	1.476e-07***
Adrenal Gland	1	54.2	23912	21981	1.844e-13***
Liver	1	38.0	23911	21943	7.127e-10 ***
Testis	1	18.6	23910	21925	1.582e-05***
Blood	1	35.0	23909	21890	3.280e-09***
Brain	1	14.5	23908	21875	0.0001425***
Lung	1	12.6	23907	21862	0.0003821***
Esophagus	1	19.8	23906	21843	8.546e-06***
Colon	1	12.9	23905	21830	0.0003299***
Stomach	1	5.5	23904	21824	0.0185580*
Thyroid	1	3.2	23903	21821	0.0756208.
Cervix Uteri	1	2.0	23902	21819	0.1569939
Blood Vessel	1	2.1	23901	21817	0.1460208
Vagina	1	1.2	23900	21816	0.2668693
Nerve	1	1.0	23899	21815	0.3103205
Pituitary	1	1.0	23898	21814	0.3151511
Ovary	1	0.6	23897	21813	0.4379597
Bladder	1	0.3	23896	21813	0.5573609
Uterus	1	0.3	23895	21812	0.5560578
Prostate	1	0.2	23894	21812	0.6973397
Spleen	1	0.1	23893	21812	0.7249901
Small Intestine	1	0.1	23892	21812	0.7569913
Breast	1	0.1	23891	21812	0.7576411
Fallopian Tube	1	0.0	23890	21812	0.9245252

Each of those comparisons is done via a likelihood ratio test. The model does not significantly improve after the inclusion of the best 17 TS as predictors (Signif. codes: 0 ‘***’ 0.001 ‘*’ 0.05 ‘.’ 0.1 ‘ ’1)

### Validation on lncRNA genes

#### Gene ontology

Due to lack of structured annotation (like GO or DO) for lncRNA, to validate our approach in this case we are forced to manually annotate them. Starting from descriptive functions reported in lncrnadb, and associating to these description GO terms, we found that the selected GO keywords rank consistently better than all the others keywords (*P*value <9*E*−06 Wilcoxon rank-sum test).

#### Disease ontology

Like in the previous case, when considering lncRNA-DO term associations from lncRNADisease we found that the selected keywords ranked consistently better than all others keywords; here however the *P*value is only marginally significant (*P*value <0.02 Wilcoxon rank-sum test). Both here and in the GO validation on lncRNA genes reported above, due to the small number of available lncRNA annotations, the results might not be as convincing as the one we perform with a similar method on the much larger set of PCG annotations. Notwithstanding these limitations, we believe it is interesting to assess in a quantitative way the performance of our algorithm directly on on lncRNA genes.

#### LncRNA implicated in cancer

We analyzed four lncRNA implicated in cancer (MYCNUT, BRAFP1, PTENP1 and TUSC7) as discussed in a recent review by Khurana et al. [27]. We highlighted the MSigDBh annotations that rank consistently at the top for all four genes. None of these functions has a significant *P*value by itself after multiple testing correction. Nevertheless, looking at the best predictions, we found many functions that are relevant in cancer such as “G2-M checkpoint”, “DNA repair’, “WNT beta Catenin signaling” [28], “E2F Targets” [29] (see Table 4).

**Table 4:** Function predicted for lncRNA implicated in cancer. Ten best MSigBDh functions are reported, none of the *P*values (Wilcoxon rank sum test) is significant per se after multiple testing correction

MSigBDh functions	Raw *P*value
E2F TARGETS	0.0029
G2M CHECKPOINT	0.0036
DNA REPAIR	0.0055
MITOTIC SPINDLE	0.006
SPERMATOGENESIS	0.0067
WNT BETA CATENIN SIGNALING	0.035
MYC TARGETS V1	0.044
HEME METABOLISM	0.093
UNFOLDED PROTEIN RESPONSE	0.13
UV RESPONSE UP	0.13

### Integration of coexpression of orthologous genes

To evaluate to what extent the gene expression in species other than human can improve functional prediction of human genes we computed FPSs for GO functions and all orthologous genes in 9 species using the Necsulea et al. gene expression dataset. Then (as before) we fit 9 bivariate logistic models that consider AS plus the data coming from one other species. We also fitted an analogous model that integrates the AS dataset from GTEx and the human data from Necsulea et al. [16]. As reported in Table 5, taken separately, each species (including homo sapiens) improves significantly the prediction when integrated with AS. It as to be noted that the bigger is the phylogenetic distance of a certain species from human, the lesser is the number of genes that can be considered since they have orthologs it that species.

**Table 5:** Log(ods) and relative *P*values associated to different single species (SSs) in logistic models

		Univariate			Bivariate			Multivariate	
Predictor	Samples	Log(odds)	AUC	*P*value	Log(odds)	AUC	*P*value	Log(odds)	*P*value
ggallus	17	–2.6	0.67	2.2e–217	–1.9	0.72	5.9e–106	–1.3	1.1e–51
ggorilla	12	–2.9	0.65	3.7e–162	–0.99	0.78	4e–19	–0.0092	0.95
hsapiens	59	–4.3	0.7	1.4e–300	–1.9	0.74	7.3e–54	–1.7	2.6e–31
mdomestica	20	–3.1	0.68	1.5e–225	–1.8	0.73	1.2e–77	–1	3.3e–23
mmulatta	14	–2.7	0.65	1e–175	–1.1	0.8	2.1e–25	0.38	0.002
mmusculus	49	–4	0.69	6.4e–275	–2.1	0.78	1.9e–74	–1.4	1.2e–24
oanatinus	19	–2.9	0.68	2.5e–229	–1.9	0.78	3.9e–92	–1.4	1.3e–46
pabelii	10	–2.8	0.64	3.7e–142	–1.2	0.81	1.7e–27	–0.53	2.2e–05
ptroglodytes	28	–3.1	0.67	1.5e–204	–1.2	0.77	1.6e–27	–0.36	0.01
xtropicalis	13	–2.7	0.65	5.5e–176	–1.8	0.78	4.4e–79	–1.5	5.3e–56

Bivariate models include two predictors: the indicated species plus AS. Note that AS and SSs derive from different expression datasets, GTEx and Necsulea respectively. The bivariate model that include hsapiens (from Necsulea) and AS shows that the contribute of hsapiens to the prediction is significant even if derive from the same specie of AS. The multivariate model consider all SSs but not AS, the AUC in this case is 0.77, the same AUC that we obtain with AS alone

### Comparison with other methods

Different computational approaches have been used for lncRNA functional predictions. Some of them rely on micro-array datasets that are biased towards the detection of protein coding genes and thus can annotate only the small fraction of lncRNA that are spotted on the array [9]. Others can infer novel annotation only for lncRNAs that are already annotated to some function [30]. The integration of other sources of information like protein-protein interactions [10] can be useful but we would like to show that our method is well suited to mine gene co-expression networks in order to perform functional prediction; we therefore choose to compare our work to the most recent similar effort by Jiang and colleagues: lncRNA2function [11]. The authors used a well established methodology: given the expression profile of a gene of interest, they perform an enrichment analysis of the genes whose expression profiles show a Pearson correlation coefficient above a fixed cutoff; the enrichment is evaluated through a Fisher exact test. The Fig. 2 shows ROC curves for lncRNA2function using different cutoffs and our approach considering only the AS predictor (without integrating tissue specific predictors or phylogenetic conservation). Since lncRNA2function is a cutoff-based approach not all the GO keyword can be scored for each gene, this is reflected in the fact that the ROC curve in this case does not cover all the range of false positive rate (FPR) but stops at a certain point that depends on the chosen cutoff. The global AUC of lncRNA2function increases if the cutoff decrease. If we consider only the region of false positive rate below 0.1 (usually the most interesting one) the partial AUC shows a maximum for a cutoff around 0.5 or 0.6. Our method outperforms the standard enrichment approach in the entire FPS range.

## Discussion

Our validation procedure on coding genes confirms the predictive power of the guilt-by-association principle. We assumed that it could be extended to lncRNAs and that the abundant functional annotation data available on protein-coding genes could be projected on lncRNAs, using gene networks built upon gene expression. This assumption will be proven extensively only when the functions of a reasonable fraction of lncRNAs will be known and well organized in systems such as GO or MSigDB. Nevertheless, by manual curation of lncRNA functions described in lncrnadb, lncRNADisease database and reviews we have shown that our approach is indeed promising.

Our method does not perform equally well for every source of information, for example it appears to work better with GO and worse with DO. More work is needed in order to investigate this fact but a contributing factor might certainly be the more abstract and complex nature of information contained in DO annotations with respect to GO terms.

Even considering only genes conserved in all species and fitting a multivariate model that evaluates all species together, we found that all species but gorilla contribute significantly. However, since we have four primates in the database, it is not surprising that the information carried by one of them is recapitulated by the others together, leading to not significant log(odds) for that species.

As expected, we found that as the number of samples considered increases the prediction performance improves; this is even more true if we consider not only all samples together but we also integrate information about tissue specificity. From this point of view it is important to increase the number of samples but also their diversity in terms of tissue of origin while considering them separately.

LncRNAs tend to be less conserved than PGCs, however nowadays more and more transcriptomic data are becoming available, even for species closely related to humans like primates, in which many lncRNA have orthologs. The approach we propose allows to exploit this data to perform lncRNA functional prediction.

## Conclusions

**Fig. 3:**
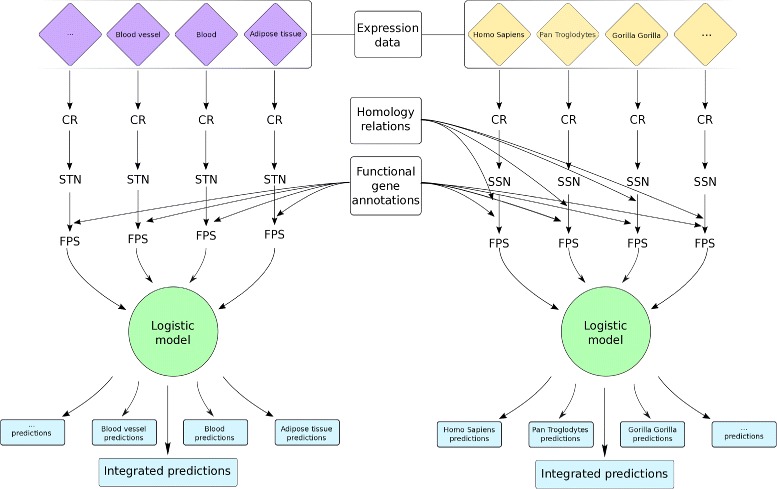
Schematic workflow of our annotation algorithm. The correlation rank (CR) among tissue- or species-specific expression profiles is used to generate complete weighted single-tissue or single-species gene networks (STN or SSN). Previously known functional annotations linked to human genes are then used along with our gene networks to compute a functional prediction score (FPS) between each gene and every annotation term; in the case of SSN it is necessary to consider homology relations between human genes and those of the considered species. The information is then combined using logistic models. The models gives a list of predictions as output; each one consists of a score associated to a gene id and an ontology term

We developed a methodology that uses gene expression data obtained from different tissues or species to predict the function of both protein coding genes (PCGs) and lncRNAs. The algorithm is based on the guilt by association principle: if a gene shows an expression profile that correlates with those of a set of genes involved in a given function, that gene is probably involved in the same function. Our approach needs a source of previous knowledge in the form of gene sets, each one associated to a keyword (e.g. GO annotations). Since coding genes are much better annotated than lncRNAs, we aim to project known functional information regarding PCGs onto lncRNAs. We propose a threshold-free algorithm (Fig. 3) able to evaluate the strength of a putative association between any gene and any keyword. It use a “gene set versus ranked list” approach that was first introduced in expression analysis through the Gene Set Enrichment Analysis (GSEA) algorithm [21]. Usually in this kind of analysis the ranked list of genes derive from a differential comparison of the expression in two conditions, instead we use gene coexpression measured in many tissues or species and produce many ranked lists, one for each expressed gene. Given a gene of interest the algorithm computes a functional prediction score (FPS) for each annotation keyword. The FPS measures the probability that a gene is associated with a keyword. Thanks to our cutoff-free algorithm, given a protein coding or lncRNA gene, we are able to evaluate all functions reported by several annotation sources in different tissues and species. Moreover, we can also query the system with a specific annotation keyword in order to obtain a ranked list of lncRNAs or PCGs that are most relevant for that term.

These analyses can be performed using our user-friendly web interface at http://www.funcpred.com/.

## Additional file

Additional file 1 Disease Ontology manual annotations. Gene-disease annotations obtained from lncRNADisease database were manually annotated to DO terms. Only lncRNA genes also contained in Ensembl are considered. (CSV 18 kb)
